# Gait in Parkinson's Disease

**DOI:** 10.1155/2019/1962123

**Published:** 2019-10-20

**Authors:** Daniel Martinez-Ramirez, Mayela Rodriguez-Violante, Adolfo Ramirez-Zamora

**Affiliations:** ^1^Tecnologico de Monterrey, Escuela de Medicina y Ciencias de la Salud, Monterrey, Mexico; ^2^Instituto Nacional de Neurologia y Neurocirugia, Ciudad de México, Mexico; ^3^Norman Fixel Institute for Neurological Diseases, University of Florida, Gainesville, FL, USA

Parkinson's disease (PD) is the second most common neurodegenerative disorder and its incidence is expected to double by 2030 worldwide. PD is characterized by a variety of motor and nonmotor symptoms caused by dysfunction of multiple interconnected brain circuits. As the disease progresses, gait and balance symptoms become increasingly problematic, greatly affecting patient's quality of life. These symptoms increase patient's risk of falls and related complications such as hospitalizations and fractures. Gait difficulties in PD are one of the most difficult symptoms to manage. While optimization of dopaminergic drugs and physical therapies are currently the main treatments, additional therapeutic interventions are needed to help patients and prevent complications. Increasing understanding of gait difficulties in PD patients is one of the greatest needs in the field.

In this special issue titled “Gait in Parkinson's Disease,” a total of 13 manuscripts were submitted, from which 5 were accepted for publication (3 experimental and 2 observational studies). The topic attracted original articles investigating a broad range of topics from clinical research to technological advances and therapies for gait difficulties in PD patients.

J. Ma and colleagues examined the working mechanism of repetitive transcranial magnetic stimulation (rTMS) on freezing of gait (FoG) by studying the sequence effect in 28 PD patients in an experimental, controlled, randomized study. The sequence effect was defined as the progressive decrease in amplitude of the sequential movements characteristic in PD patients. Patients received either real or sham 10-Hz rTMS over the supplementary motor area for ten sessions over two successive weeks. Results of the primary outcome showed that rTMS did not improve the sequence effect. However, a transient beneficial effect was observed on FoG and other gait parameters including ambulation time, cadence, step count, and velocity gait. The authors conclude that other mechanisms of how rTMS works, besides improving the sequence effect, should be explored. This study has in the experimental design its most important strength. In addition, this is the first study investigating the role of rTMS on sequence effect. Still, several important limitations must be considered including the study sample size. Research investigating the effects of rTMS in FoG began over a decade ago and continues to be an active field in neuromodulation [1]. A recent meta-analysis by Y. W. Kim et al. reported significant improvement in the freezing of gait questionnaire (FOG-Q) scores, but no differences in the Unified Parkinson's Disease Rating Scale (UPDRS) scores with rTMS when compared with placebo [2]. Evidence supports the role of rTMS in the treatment of PD, but further studies are required to elucidate and determine its mechanism of action, the optimal stimulation site, stimulation parameters, and duration and number of stimulation sessions [3–7].

R. Marsh and colleagues studied the benefits of visual cueing on FoG in an experimental uncontrolled study of 20 PD patients. During a two-minute walk and an obstacle course, results showed an improvement in distance walked during the two-minute walk test when a cueing device was on phasic and tonic modes. The tonic visual cueing demonstrated superiority over the phasic visual cueing. However, this benefit was not observed during the obstacle course. Although different wearable visual cueing devices have shown improvement on FoG episodes in PD patients [8], few studies have studied the effect of different modes of visual cueing. The strength of the study is the novel approach used to experiment with two patterns of visual cueing, phasic and tonic patterns. However, not having a control group and sample size are important limitations to consider. Further studies are required to clarify the role of different visual cueing modes on FoG.

K. Sato and colleagues studied the clinical benefits of rehabilitation after DBS surgery. An experimental uncontrolled study was planned to examine the effect of two weeks of rehabilitation on gait and postural instability of PD patients following STN-DBS surgery. Sixteen patients were analyzed retrospectively. Rehabilitation was focused on muscle strengthening with stretching and balance training. Results showed an improvement in balance measured by Mini-BESTest and gait measured by Timed “Up and Go” (TUG) test when compared to baseline evaluations. The role of rehabilitation therapies shortly after deep brain stimulation (DBS) surgery is unknown, since most evidence focuses on longer term management. This study adds to a better understanding and timing of the role of PT after DBS for PD or other movement disorders. However, the uncontrolled design of the study and sample size are important limitations to consider. Recently, N. Allert et al. discussed and highlighted the importance of a coordinated therapy within a multidisciplinary team to achieve maximal results after DBS therapy. Still, guidelines in the postoperative rehabilitation management of these patients are required [9].

B. Muñoz-Ospina and colleagues studied the effects of aging in gait in an observational cross-sectional comparative study using the Microsoft Kinect sensor camera in 30 PD patients compared with 30 age-matched controls. Results demonstrated that PD patients exhibited prolonged swing and stance times and lower speed values compared to controls. However, this was not observed in the group of 76–88 years old. The authors concluded that the consequences of age in gait of PD patients should also be considered when approaching these patients. The strength of the study is the use of 3D gait analysis in patients compared to controls; however, the cross-sectional design of the study is a limitation to consider since a cause-effect relationship is not possible to consider. Despite the study design, the authors provide reasons to be optimistic in the use of technology to better analyze gait in PD patients. This can increase our understanding and knowledge on how to focus therapy [10].

Finally, C. Geroin and colleagues assessed the effects of axial deformities such as camptocormia on gait. The authors conducted an observational, cross-sectional comparative study to compare gait parameters, gait variability, and asymmetry and postural control of 46 PD patients with and without camptocormia. The study demonstrated that lower trunk camptocormia was associated with more severe gait and postural impairment. PD deformities are important to consider when analyzing and providing therapy for gait problems. Recent studies using 3D gait analysis have shown reduced movements in the hip and knee joints of patients with camptocormia [11]. The present study provides important information with regards to postural deformities of the lower trunk and its possible negative impact on gait. However, studies with better design are required to establish a more direct cause-effect relationship. Despite these limitations, results suggest that technology can be used to better define camptocormia in order to provide individualized therapies.

In summary, we hope that this special issue brings new insights into the latest advances in the diagnosis, treatment, and pathophysiology of gait difficulties in PD. We hope this new information will help other researchers pave the way for the development of strategies to help PD sufferers.
